# Maternal and perinatal guideline development in hospitals in South East Asia: the experience of the SEA-ORCHID project

**DOI:** 10.1186/1478-4505-7-10

**Published:** 2009-05-08

**Authors:** Tari J Turner, Jacki Short, The SEA-ORCHID Study Group

**Affiliations:** 1 Monash Institute of Health Services Research, Monash University, Monash Medical Centre, Clayton, Victoria, Australia; 2 Perinatal Services Network, University of Sydney, Sydney, NSW, Australia

## Abstract

**Background:**

Clinical practice guidelines (CPGs) are commonly used to support practitioners to improve practice. However many studies have raised concerns about guideline quality. The reasons why guidelines are not developed following the established development methods are not clear.

The SEA-ORCHID project aims to increase the generation and use of locally relevant research and improve clinical practice in maternal and perinatal care in four countries in South East Asia. Baseline data highlighted that development of evidence-based CPGs according to recommended processes was very rare in the SEA-ORCHID hospitals. The project investigators suggested that there were aspects of the recommended development process that made it very difficult in the participating hospitals.

We therefore aimed to explore the experience of guideline development and particularly the enablers of and barriers to developing evidence-based guidelines in the nine hospitals in South East Asia participating in the SEA-ORCHID project, so as to better understand how evidence-based guideline development could be facilitated in these settings.

**Methods:**

Semi-structured, face-to-face interviews were undertaken with senior and junior healthcare providers (nurses, midwives, doctors) from the maternal and neonatal services at each of the nine participating hospitals. Interviews were audio-recorded, transcribed and a thematic analysis undertaken.

**Results:**

Seventy-five individual, 25 pair and eleven group interviews were conducted. Participants clearly valued evidence-based guidelines. However they also identified several major barriers to guideline development including time, lack of awareness of process, difficulties searching for evidence and arranging guideline development group meetings, issues with achieving multi-disciplinarity and consumer involvement. They also highlighted the central importance of keeping guidelines up-to-date.

**Conclusion:**

Healthcare providers in the SEA-ORCHID hospitals face a series of barriers to developing evidence-based guidelines. At present, in many hospitals, several of these barriers are insurmountable, and as a result, rigorous, evidence-based guidelines are not being developed. Given the acknowledged benefits of evidence-based guidelines, perhaps a new approach to supporting their development in these contexts is needed.

## Background

Clinical practice guidelines (CPGs) are commonly used to support practitioners to improve practice. Guidelines are "systematically developed statements to assist practitioner and patient decisions about appropriate health care for specific clinical circumstances"[1]. Evidence-based guideline development emphasises the importance of linking guideline recommendations to the scientific evidence that supports them, identified through a rigorous systematic identification and appraisal of all relevant research. Evidence-based guidelines should be developed by a multidisciplinary group which includes both clinicians and consumers. This group considers the best available research evidence and uses this as the basis of their recommendations. Where there is no relevant research evidence, recommendations are based on the consensus of the group.

Despite the fact that development of evidence-based guidelines is increasing [2,3] and there is a standard process for guideline development [4], which has been described in several published guides such as those produced by the Australian National Health and Medical Research Council, the UK National Institute for Health and Clinical Excellence, the New Zealand Guidelines Group and the Scottish Intercollegiate Guideline Network [5-8], many studies have raised concerns about the quality of recently developed guidelines [9-14]. Methodological issues have also recently been highlighted in a review of use of evidence in World Health Organization guidelines [15].

The reasons why guidelines are not developed following the established evidence-based guideline development methods are not clear, though the recommended process is extremely time and resource-intensive [3,16-18] and requires a specific set of skills in identifying, appraising and synthesising research.

The South East Asia Optimising Reproductive and Child Health in Developing Countries (SEA-ORCHID, ) project is a five-year collaborative project between Thailand, Malaysia, the Philippines, Indonesia, and Australia. By establishing a network of researchers and teachers of evidence-based health care in nine hospitals, across four South East Asian countries, supported from Australia, SEA-ORCHID aims to increase the generation and use of locally relevant research and improve clinical practice in management of pregnancy and childbirth. SEA-ORCHID is jointly funded by the Wellcome Trust and the Australian National Health and Medical Research Council.

**Figure 1:**
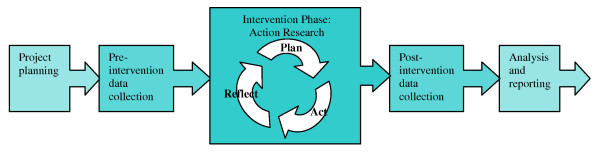
**The stages of the SEA-ORCHID project**.

The SEA-ORCHID project consists of five stages; pre-study, pre-intervention data collection, intervention, post-intervention data collection, and reporting and dissemination. Details of the project methods and pre-intervention audit of clinical practice have been published previously [19,20] see Figure 1.

The evaluation of the SEA-ORCHID project includes quantitative evaluation of change in clinical practice, health outcomes, research activity and guideline development or adaptation combined with qualitative investigation of the barriers to and enablers of these changes.

Quantitative data from the baseline assessment in the SEA-ORCHID project indicated that development of evidence-based guidelines according to recommended processes, or adaptation and implementation of existing evidence-based guidelines was very rare in the SEA-ORCHID hospitals. As is reported in more detail elsewhere [21], only a small number of CPGs based on systematic searches for evidence and multidisciplinary involvement had been developed at national or regional level and were locally implemented. There were a few evidence-based guidelines available at some of the hospitals and most hospitals had a wide range of non-evidence-based local protocols.

The SEA-ORCHID Investigators suggested that there were aspects of the recommended evidence-based guideline development or adaptation process that made it very difficult, or not feasible, in the participating hospitals. As a result of these barriers, the Investigators thought that it was unlikely that substantial progress would be made in guideline development within the SEA-ORCHID project.

We therefore aimed to explore the experience of guideline development and particularly the enablers of and barriers to developing and adapting evidence-based guidelines in the nine hospitals in South East Asia participating in the SEA-ORCHID project, so as to better understand how evidence-based guideline development and adaptation could be facilitated in these settings.

## Methods

During the final nine months of the intervention phase of the SEA-ORCHID project (July 2007 – February 2008), semi-structured, face-to-face interviews were undertaken with healthcare providers (nurses, midwives, doctors) from the maternal and neonatal services at each of the nine participating hospitals.

The interviews were conducted by two Australian SEA-ORCHID team members, a psychologist (JS) and a health services researcher (TT). The interviewers did not have clinical experience in the research area, but both had a background in evidence-based practice and research methods. JS had experience in supporting behavioural change from a psychological perspective and TT had experience implementing evidence-based change in a clinical setting and substantial experience in developing evidence-based guidelines. Where possible the interviews were conducted with both interviewers present, however, where made necessary by time limitations, interviews were conducted by one or other of the interviewers.

### Participants

Participants were healthcare providers working in the maternal and neonatal areas of the SEA-ORCHID hospitals. At each hospital we aimed to interview two junior and two senior doctors, and two junior and two senior nurses or midwives, as relevant to the hospital structure. Interviewees included all levels of clinicians, including those responsible for patient care and organisational policy setting. We had hoped to interview consumers however this was not covered by our ethics approval.

We also interviewed healthcare providers who had undertaken evidence-based practice training fellowships offered at the Australian SEA-ORCHID hospitals during the course of the project and the members of the SEA-ORCHID team at each of the hospitals.

Interviews were most often conducted with individuals or pairs of healthcare providers, however a small number of group interviews were conducted.

### Identification and consent

SEA-ORCHID personnel were asked to identify potential participants at each of the sites. Selection was largely pragmatic, based on availability for interview. Potential participants received an explanatory statement and signed a consent form to indicate informed consent. In Malaysia and the Philippines, explanatory statement and consent forms were provided in English, and interviews were conducted in English. In Indonesia and Thailand, the explanatory statement and consent forms were translated into the local languages and interpreters were used for the interviews.

### Ethics approval

Ethics approval was granted by the University of Sydney, Monash University and the relevant ethics committees in each of the South East Asian countries.

### Description of questions

Interviews were loosely based on a pre-specified interview protocol, but the detailed questions asked varied according to the level of experience and interest, and previous responses of the interviewees. The questions were designed to explore the interviewees' awareness and experience of undertaking evidence-base practice, doing research and developing guidelines or protocols. In this paper we report the results relating to development of guidelines or protocols.

### Analysis

Interviews were audio recorded, transcribed and de-identified, and the data analysed thematically using NVivo software. Data relevant to guideline development were analysed in emerging themes based around the value, product and process of guideline development.

## Results

Seventy-five individual, 25 pair and eleven group interviews were conducted in the nine SEA-ORCHID hospitals. In a small number of hospitals we were unable to interview the planned number of staff as there were insufficient staff employed and/or available. In other hospitals more interviews were conducted because of wider interest.

Participants had a wide range of levels of experience with evidence-based guideline development. Very few nurses or midwives had personal experience of developing evidence-based practice guidelines. Familiarity with the process was more common among doctors, and particularly senior doctors. Several senior doctors had attempted, or were currently attempting, to write guidelines and a small number were very experienced, having produced evidence-based guidelines at a local, regional and national level. Many of those who were currently involved in evidence-based guideline development had been taught or supported to do this through the SEA-ORCHID Project. Most interviewees had some experience of developing guidelines in their units, following non-evidence-based methods.

Responses were grouped into emerging themes as described below.

While there was wide variation between hospitals in levels of resourcing, organisational structure, number of deliveries etc, the results of the interviews were remarkably consistent across hospitals and countries. Particular thematic differences between hospitals are noted in the text.

Themes emerging from analysis:

THE VALUE:

The usefulness of guidelines

THE PRODUCT:

Use of evidence in guidelines

Format of guidelines

THE PROCESS:

Time consuming and difficult

Lack of awareness of process

Scope of guidelines

Adapting existing guidelines

Searching for evidence

Arranging guideline development group meetings

Multi-disciplinarity

Consumer involvement

Achieving consensus

Importance of keeping guidelines up-to-date

### THE VALUE

#### The usefulness of guidelines

Most interviewees believed that guidelines could be useful in staff training, outlining the process of care, keeping practice up-to-date with research and standardising practice.

If you have guidelines everybody does the same thing and do the standard thing. Junior Nurse

Some clinicians, however, expressed doubts about whether guidelines were being used.

Q: Do you think guidelines are useful?

*A: It is useful if you, if most of us read them*.

Q: Do you think they are used?

A: I don't think so. Senior Doctor

Q: So how often would you... look at a policy or a guideline?

A: Honestly?

*Q: Yeah, honestly*.

A: Not at all. Senior Nurse

### THE PRODUCT

#### Use of evidence in guidelines

Almost all interviewees believed that it was important to base guidelines on evidence.

First a good guideline for me is one that is based on research and good research that has been verified and the bad ones would be those that are not based on valid research. Junior Nurse

It is important because basically if it is based on the evidence previously tested in studies then we know, ... we can have proof to the patient that we have done our best. Junior Doctor

Many noted, however, that the guidelines they developed, or those developed by others, were not based on research evidence.

The guidelines are still, in most hospitals, especially in the local hospitals outside the centre of education, I think from the experience are based on textbooks, according to what they've got when they are residents. Senior Doctor

#### Format of guidelines

Interviewees thought that the format of guidelines was very important. However, two very different thoughts were expressed by interviewees about the ideal format of guidelines: that they must be simple and quick to read and that they should be detailed and thorough.

It should be short and easy for read. Junior Doctor

I think a good guideline should be very specific, in detail, so that everybody can read it and everybody can understand it more. Senior Doctor

Interviewees also suggested that a diagram or flowchart was a very useful format for a guideline.

Definitely the flowchart because it is easier to understand and faster. Junior Doctor

### THE PROCESS

#### Time consuming and difficult

One of the recurring barriers to development identified in the interviews was how difficult and time consuming the process of guideline development was when undertaken rigorously. Many interviewees mentioned guideline development methods that were designed to meet the AGREE (Appraisal of Guidelines Research and Evaluation) Instrument [22] criteria, the most widely accepted tool for appraising the quality of guidelines.

Making a guideline from scratch is a whole lot of work, ...according to the book it takes it's a process of years. Senior Doctor

Because the full guideline with AGREE tool, I think if I want to develop the guideline for breech presentation I have to, ... make the question for the patient and then I have to search for the literature for each question and then I have to approve into the recommendations and level of evidence or grading of recommendation and then I have to send this draft to the committee who develop the guideline and then make it a full version. So it's just really, really long way. Senior Doctor

#### Lack of awareness of process

Some interviewees identified that there was a lack of awareness or acceptance of the process of developing evidence-based guidelines among their colleagues, and that this was a barrier to developing high-quality guidelines.

There is a society for all the neonatologists in [our country], and they had a weekend meeting, and they wanted us to do a guideline right then and there. ... And so they have no idea what is really involved. So I think that's the barrier, that's one of the barriers here, that people don't really know what the guidelines means. Senior Doctor

#### Scope of guidelines

Some interviewees noted that the scope or breadth of the guidelines had a substantial impact on the amount of work involved in developing the guideline.

So I think that why it takes a long time is because of the scope. Senior Doctor

#### Adapting existing guidelines

Several of the interviewees noted that they adapted guidelines for use in their hospitals based on those produced in other settings.

So I think our guidelines are more adaptations from, more than guidelines, it's not like we start from scratch to develop them. Senior Doctor

Several different concerns were expressed about the difficulty of adapting externally generated guidelines for use in SEA-ORCHID hospitals. These included relevance of the existing guideline to the new setting, methodological concerns about the process of adapting guidelines, and availability of guidelines to adapt.

##### Relevance

Relevance of guidelines was a central concern. Differences in patient population, in culture, economic status and availability of equipment and infrastructure were all reported to reduce the relevance of guidelines developed in other settings.

Many of our protocols are quite heavily based on RPA [Royal Prince Alfred Hospital, Sydney] protocols as well, and we have some national guidelines, and all of them need some scrutiny because [the local area] is quite different from the rest of the country actually. ... Our culture is quite different and our economic status is quite different. Senior Doctor

##### Methodological concerns

Among those clinicians who accepted the concept of adapting existing guidelines, and had found relevant guidelines, some had concerns about the validity of methods for adapting guidelines.

The process [of adapting a guideline] also I think maybe easier but we have to have a good methodology to do this. ... [Can] you put the reference to this just only one this guideline or you have to look the reference from the good guideline already and then put it into your guideline? ... – He has done already but you copy on your own, is not good, I think. Senior Doctor

Interviewees also noted that following established methods for appraising the quality of guidelines before adapting them was also very time consuming.

No, we have not used the AGREE tool to appraise them because I think even that takes a long time. Senior Doctor

##### Availability

Two barriers were identified as limiting the availability of guidelines to adapt. The first was an access issue; in some SEA-ORCHID hospitals clinicians reported that they were very limited in being able to access recent guidelines. The second issue was the lack of existing evidence-based guidelines in areas of interest.

I believe that we can apply these guidelines to the situation here, but one of the problems that I encounter is it's very difficult for us to actually get the guidelines because of access problems and sometimes we get guidelines that's not updated. Junior Doctor

*A: What we are using now are the American guidelines. But many of the points there aren't really applicable here. So adapting a new one*.

Q: Are the guidelines here that you're adapting, are they evidence-based guidelines?

A: That's it, they're not. Senior Doctor

#### Searching for evidence

Interviewees noted that searching for evidence to underpin their guidelines was of fundamental importance to the development process, but also that it was very time consuming.

The search for literature is very important because we have to get the most updated, the highest level of evidence. That takes time. Senior Doctor

Some interviewees explained that they did not undertake a systematic review of the literature as recommended by accepted methods of guideline development, but took a more pragmatic approach.

A few papers, probably RCTs or guidelines from books, from journals ... five to six references for each chapter – that's all what we do. Not an extensive literature review on that topic. Senior Doctor

Other interviewees noted that they were limited in their ability to carry out searches for research evidence because of limitations on availability of computers, internet access and full text journal articles.

We have internet access here, but our computer is non-functional right now. ... It's, we have only one computer for that area, ... we're using only one for about 50 people actually. Junior Doctor

The electronic resources is not very reliable. There is but sometimes it's running very slow ... and people don't want to sit there very long in front of the computer on the internet so that is also one of the challenges we have to face so not only the skills but also the resources itself is not very adequate. Senior Doctor

Though not often linked directly to guideline development, participants, and particularly nurses, frequently noted that lack of skill in English and lack of skill in using computers and the internet limited their ability to search for evidence

I do this to search from website Cochrane Library but I have the problem with English, it's a little bit difficult for me to read. Junior Nurse

Well, the SEA-ORCHID project has already given us great facilities, like the computer for internet, for browsing. But unfortunately we have such lack of capacity in actually using the internet. Senior Nurse

#### Arranging guideline development group meetings

Interviewees highlighted that there were basic hurdles with the guideline development process that could not easily be overcome. One of the most frequently mentioned was the need to arrange meetings of those involved in guideline development.

So if you want to meet with the four of us, it's almost impossible to have everybody free at one time. We can make a minimum of three, but ... it's so difficult actually, sometimes they have something on, and then we have something on, and it gets postponed. And it dies off. Senior Doctor

We're at a standstill now, we're still in review of literature, we haven't been able to move on, it's so hard to get everyone together. Senior Doctor

#### Multidisciplinarity

Having a multidisciplinary group responsible for guideline development, as is recommended by evidence-based guideline development methods, was not usual practice at most SEA-ORCHID hospitals. Most often the guidelines were largely developed by the senior medical staff.

Q: Are nurses involved in writing the protocols?

A: No. Consultants. Consultants and the specialists they are the ones who go in a closed room I think. ... Then they come up with the protocol. Senior Nurse

Even the nurses, they are not easily involved. I mean, it's usually the doctors who develop it. It's because it's the doctors who make the orders. So it's usually those who develop it. Senior Doctor

In some hospitals this was beginning to change.

I won't say that all guidelines, but most guidelines will actually involve the nurses because in the past there was only the doctors would decide. But recently the nurses contribution and this is slightly more. Senior Doctor

And in some hospitals it was usual practice to involve nurses and midwives in some aspects of the guideline development process.

Before we started the development of CPG we invite all the midwives and also nurses to have a meeting with us and then we have a brainstorming about any question about pre-term delivery in your mind. Senior Doctor

#### Consumer involvement

None of the interviewees had experience in involving patients or their families or carers in the development of guidelines, as is recommended by evidence-based guideline development methods.

We don't usually involve the consumers.... During our stay in Australia, we had a visit with a group of consumers, and when I realise that we have to involve the consumers in developing guidelines, that unfortunately we don't easily involve them. Senior Doctor

Some interviewees were very uncomfortable with the idea of involving consumers in guidelines development. These interviewees were all less experienced with the guideline development process.

I don't think it's very practical, and it's not very safe. Because what patients know and what patients want do not always correspond to what is the best practice at that point in time. Junior Doctor

Other interviewees thought that there might be a role for consumers, but felt that there were substantial limitations on that role.

Q: What do you think about consumer involvement in a hospital setting?

*A: Ideally that would be great*.

Q: But?

A: But I don't think it might be applicable for all aspects of the CPGs. For example, I think it will be applicable if the topic was enema or shaving or episiotomy even, but I don't know how much influence they can have on what type of suture I have to use for caesarean section or what's the best antibiotic that I have to use or whether I have to use CPAP or mechanical ventilation. So it's more applicable for some issues and topics, but not all. Senior Doctor

#### Achieving consensus

The process of achieving consensus on the recommendations of the guideline was felt to be very important, especially in terms of enabling the later implementability of the guideline, but also a potential barrier to development if agreement could not be reached.

*A: In our case, we are stopping the guidelines because there have been some problems *...

Q: So you've had to stop for a while, is it for clinical reasons? Because you were too busy or?

A: No, not the clinical reason [laughs]...I think it's more on conflicting ideas. Junior Doctor

#### Importance of keeping guidelines up-to-date

One of the clearest messages from interviewees was that guidelines should be regularly updated.

One thing that can improve about babies' management, I think we should routinely upgrade the protocol. Junior Doctor

Regularly updated guidelines were seen to support evidence-based practice, and to enable change in line with new evidence.

I think in [our department], changing the practice is not very hard because we are always updating our protocol, if we think that it is not up-to-date any more. Senior Doctor

Outdated guidelines were seen to be potentially detrimental, particularly if clinicians were diligent in following guidelines.

Well it's very difficult because you know everybody here abides by the guidelines. But then some of these guidelines are not up to date. You know, we have a lot of new information, we have a lot of new practice, the correct practice, but it's not updating the guidelines. Whereas people follow the guidelines, so... Junior Doctor

One interviewee noted that what was need was a regular updating process, with dedicated clinicians to maintain the guidelines.

With these guidelines is that some of them are outdated already, so they have to be updated. So there has to be a committed set of people who will – like in Cochrane, that they have updates periodically. Senior Doctor

## Discussion

Almost all the healthcare providers interviewed in these hospitals were positive about the potential for guidelines to improve and standardise practice – at least in theory. Interviewees were less confident that the guidelines were achieving their potential, largely as the result of two factors, barriers to the development of guidelines and barriers to the use of guidelines. These results echo those of other studies in similar settings. For example, a study of facilitators and barriers to the adoption of evidence-based perinatal care in Latin America found that guidelines were both valued and felt to be necessary but were not enough to change practice on their own (Belizan 2007).

Healthcare providers in our study were also clear that guidelines should be developed on the basis of the best available research evidence, but expressed strong concerns about the feasibility of the current methods to achieve that in their setting. The substantial time investment required to find evidence and lack of access to the internet and evidence sources, were repeatedly mentioned as barriers to this process. Limitations in skill in finding and appraising evidence for use in guidelines were mentioned only in passing, however this may reflect the fact that most interviewees had not undertaken the systematic reviews of research required by the established guideline development methods, and were unaware of the skills required. Lack of awareness of the development process or lack of acceptance of an evidence-based framework to clinical practice were also noted barriers.

Arranging times for the guideline development group to meet was a major barrier to guideline development. This is particularly interesting given that at most sites, these meetings were only of the senior medical staff. Involving nurses or midwives, junior staff and consumers would be likely to make arranging meetings only more difficult. Wider representation may also complicate the process of achieving consensus, which several clinicians reported was already very difficult.

One of the strongest messages from the interviews was the central importance of keeping guidelines up-to-date with advances in research. While acknowledging the work involved, interviewees felt very strongly that this should be a priority.

There are clearly substantial barriers to developing evidence-based guidelines in the SEA-ORCHID hospitals. The extensive time commitment required for systematic reviews, need for reliable access to research evidence and difficulties in arranging guideline development group meetings were fundamental problems that stopped interviewees from developing evidence-based guidelines. While adapting existing guidelines can be a useful approach, clinicians report that it is not always possible or appropriate. Issues like multidisciplinary representation and involvement of consumers were also reported as being difficult to address. If evidence-based guidelines are going to be developed or adapted and implemented in these resource-poor environments, perhaps a more pragmatic approach is needed. Such an approach might limit the process of searching for evidence to a small number of reliable sources of high quality evidence, require a less intensive multidisciplinary consultation process, or focus on a smaller number of priority clinical questions with the potential to more significantly impact to on clinical practice, rather than aiming to address an entire clinical area. It would be interesting to develop and test a simpler, more practical approach to guideline development and compare the results of this new approach against the established rigorous processes. Alternative strategies such as financial incentives or regulatory requirements for hospitals to have guidelines might also be beneficial in increasing the development of guidelines. Similar research in developed country settings would also provide useful information on to what extent the experience of guideline development is common across settings.

This study has a number of limitations. Interviewees were selected on a largely pragmatic basis and their views may not be representative of all of their colleagues. However the healthcare providers interviewed provided a wide range of views on the issues discussed. Only a minority of the healthcare providers interviewed had in-depth experience with the evidence-based guideline development process, and those who were familiar with the process were more likely to be experienced and from a medical background, so these results may overemphasise the views of these groups of clinicians. However the fact that junior clinicians and nurses or midwives were less likely to have been involved in guideline development highlights one of the aspects of the recommended guideline development process which is not being followed at many of the SEA-ORCHID sites, a guideline development group that includes representation from all relevant professional disciplines, levels of seniority and consumers.

## Conclusion

Healthcare providers in the SEA-ORCHID hospitals face a series of barriers to developing evidence-based guidelines. At present, in many hospitals, several of these barriers seem insurmountable, and as a result, rigorous, evidence-based guidelines are not being developed. Given the acknowledged benefits of evidence-based guidelines, perhaps a new approach to supporting their development in these contexts is needed.

## Competing interests

The authors declare that they have no competing interests.

## Authors' contributions

TJT conceived of the project, undertook the analysis and prepared the first draft of the article. TJT and JS developed the methods, undertook the interviews and revised subsequent drafts. Both authors read and approved the final manuscript.
